# Suspected spread of hepatitis A virus from a restaurant among adults in rural area of the Kerala state, India

**DOI:** 10.1017/S0950268819000967

**Published:** 2019-05-29

**Authors:** Y. K. Gurav, G. Retheesh Babu, K. P. Vinu, K. S. Lole

**Affiliations:** 1 ICMR-National Institute of Virology, Pune, India; 2 IDSP, District Health Office, General Hospital Campus, Ernakulam, Kerala

**Keywords:** Hepatitis A

## Abstract

India is experiencing a substantial decrease in early childhood exposure to hepatitis A virus (HAV). Kerala has experienced several hepatitis A outbreaks in young adults/adults in the recent past. The current hepatitis outbreak occurred in Nellikuzhi, Kerala state, India in December 2016. Investigation was carried by preparing a line list of suspected hepatitis cases. The blood and stool samples collected from patients were tested for anti-HAV/anti-Hepatitis E virus (HEV) immunoglobulin (IgM) antibodies and RNA respectively. A total of 562 suspected hepatitis cases were reported during the outbreak. Along with the first case (35 years, male), 86.1% (484/562) of the cases gave history of consuming food/water/cold drinks from one restaurant. Anti-HAV IgM positivity was 74.5% (73/98) in tested samples and amongst the positives, 81% were adults/young adults and adolescents. None of the samples tested positive for anti-HEV IgM. There were three HAV associated deaths without any co-morbidity. Sequence analysis of HAV RNA positive stool samples showed the presence of genotype IIIA HAV. The suspected source of the infection was a private well situated in the premise of a restaurant. Considering increasing HAV naive population in Kerala, there is a need to introduce hepatitis A vaccine in high-risk age groups.

## Introduction

Hepatitis A virus (HAV) causes a self-limiting disease of the liver. The virus is transmitted via a faeco–oral route through contaminated water, food or person-to-person contacts. In developing countries, a majority of children (>90%) are exposed to HAV during early childhood and develop lifelong immunity [1]. India is passing through a changing epidemiological phase for hepatitis A. Though India is still endemic for the virus, with improvement in economic and living conditions, HAV exposure of children is decreasing [2]. HAV naïve individuals thus remain susceptible to the infection in the later phase of life. Majority of HAV infections in children are asymptomatic or with mild symptoms, while, infections in young adults/adults are with noticeable symptoms. India has seen increase in both sporadic cases as well as outbreaks of hepatitis A among adults in the recent past [2, 3]. Kerala state in the southern India has experienced alarmingly large number of hepatitis A outbreaks (*n* = 84) [4], mostly in young adults/adults, during last 5 years [3–6]. In December 2016, the ICMR-National Institute of Virology (NIV), Kerala unit was approached by the district health authorities after observing unusual rise in hepatitis cases among adults around Nellikuzhi, a suburban area of Kothamangalam town, Ernakulam district, Kerala state. Outbreak investigations were carried out jointly by the NIV team, district health authorities and Kerala state health authorities. The objective of the outbreak investigation was to confirm the aetiological agent and to characterise the outbreak by time place and person distribution.

## Materials and methods

### Study area and population

Nellikuzhi Panchayat has a population of 47 679 (23 898 males and 23 781 females). The panchayat area is divided into 21 wards with ~11 734 houses. For the outbreak investigation, the study population included persons living in the Nellikuzhi panchayat and adjoining nine panchayat areas (Pindimana, Kottapady, Varpetty, Assamannur, Pallarimangalam, Kuttampuzha, Paypra, Kavalangad and Vadavucode) in the Ernakulam district.

### Epidemiological and clinical investigations

A suspected case of acute hepatitis was defined as the person presenting with icterus or dark coloured urine along with one or more symptoms such as fever, loss of appetite, fatigue, right upper quadrant tenderness and vomiting. Clinical and epidemiological details of each suspected case were collected in the predesigned proforma. History of travel and consumption of food/water from outside sources were noted. A line list of suspected hepatitis cases was prepared. Cases registered with the primary health centre (PHC) Cheruvattor, private practitioners and traditional healers practicing in the affected area during the investigation period (December 2016–March 2017) were also considered for inclusion in the line list and duplicate entries were removed. Examination and interviews of representative suspected cases were carried out in the outpatient clinic of the PHC, tertiary care hospitals and households in the Nellikuzhi.

A sanitary survey was conducted in the affected area to identify possible source of the virus. Geographical information system (GIS) tools in Epi-Info version 7.2.2.6 were used for capturing geo-coordinates of the laboratory confirmed hepatitis cases and preparing map graphics. To know the distribution pattern of the cases and to calculate the nearest neighbour index (ratio of the observed distance divided by the expected distance between two nearest points) and *Z* score as a measure of statistical significance, nearest neighbour analysis algorithm, QGIS version 2.18.16 was used.

### Specimen collection, serosurvey and serological tests

Blood samples (2–5 ml) were collected randomly from representative suspected hepatitis cases from the Nellikuzhi and adjoining areas by the health centre and sent to the public health laboratory, Ernakulam. Investigating team also collected blood samples randomly from the suspected hepatitis cases reporting at the health centre and those admitted in the St. Joseph Hospital, Kothamangalam, located ~10 km away from the Nellikuzhi during the investigation period. We tested, samples collected by us and samples stored in the public health laboratory, Ernakulam for anti-HAV immunoglobulin (IgM) (anti-HAV IgM ELISA kit, General Biologicals, Taiwan) and anti-hepatitis E virus (HEV) IgM antibodies (in-house ELISA kit) [7]. Stool samples were collected randomly from a representative number of confirmed anti-HAV IgM positive cases for detecting the virus.

A serosurvey was conducted in the affected area (ward no. 13, Nellikuzhi), where individuals were likely to be affected by the current outbreak and in the unaffected area, Ayavana village, 20 km away from Nellikuzhi, having a total population of 21 224 (10 732 males; 10 492 females), as the demographics of these two communities were matching. Serosurveys were conducted to know baseline exposures of these populations to HAV and HEV. For that, we involved local community leaders and health care providers and adopted a convenient sampling method. This did not involve any age and sex matching with the cases. Blood samples were collected by the trained staff at the health centre from all age groups except for the age group of <5 years due to concerns of the parents, after obtaining written consent from the participants. The blood samples from serosurvey subjects were tested for anti-HAV IgM and IgG using ELISA kits (General Biologicals, Taiwan) and for anti-HEV IgM and IgG using in-house ELISA kits [7].

### Water samples

Water samples were collected from wells, tanks, households and restaurants in the affected area and processed for HAV RNA detection. Water samples were also tested for faecal coliform bacteria in the Regional Public Health Laboratory of Ernakulam.

### Polymerase chain reaction, sequencing and phylogenetic analysis

Stool samples were processed for preparing 10% suspensions in 1× phosphate buffered saline, and used. Water samples were directly used for HAV RNA detection. Detection of HAV RNA, sequencing and phylogenetic analysis were performed as described earlier [2].

## Results

### Epidemiological and clinical findings

**Fig. 1.**
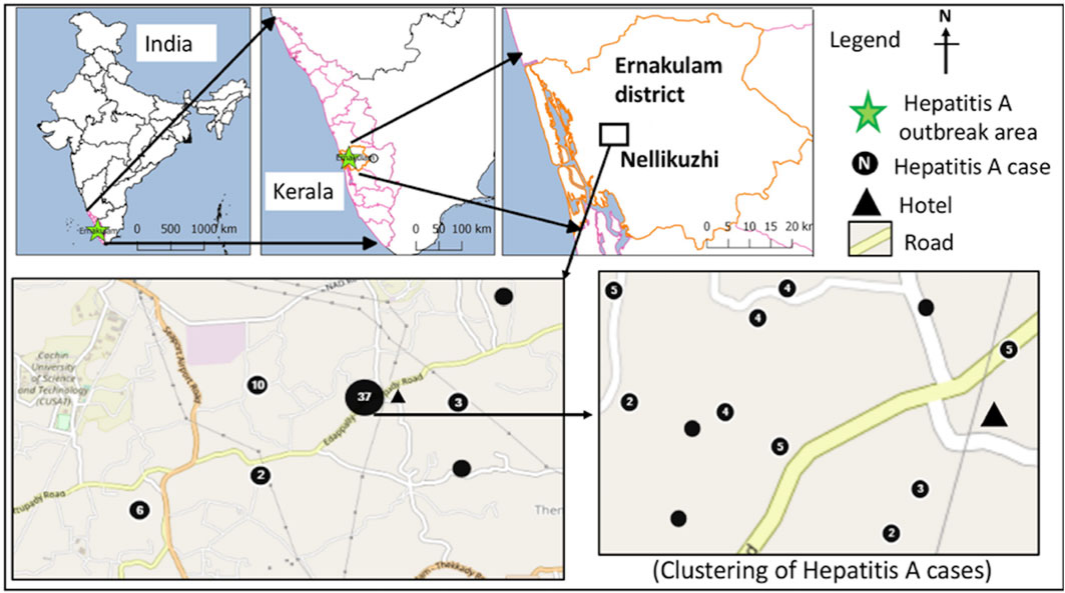
Distribution of laboratory-confirmed hepatitis A cases (*n* = 60) in the study area during the investigation period from October 2016 to January 2017.

A sudden rise in the number of suspected hepatitis cases was noticed by the tertiary care hospital, St. Joseph Hospital, Kothamangalam, Ernakulam district during the mid-November, 2016 (Fig. 1). Local health authorities and NIV's Kerala unit were informed about the possible outbreak. The District health authorities immediately initiated investigations, line listed the suspected hepatitis cases and implemented preventive and control measures in the affected area. Of the total 562 suspected cases (age 4–68 years), 75% (422/562) were from the Nellikuzhi grampanchayat. Majority (67.6%, 380/562) of these were young adults (20–39 years); 19.4% (109/562) were adolescents (10–19 years) and 2.8% (16/ 562) were children (⩽9 years). Laboratory testing of 98 blood samples from the suspected cases showed anti-HAV IgM positivity in 74.5% (73/98). Among IgM positives, 61.6% (45/73) were young adults, 19.2% (14/73) were adolescents and 8.2% (6/73) were children. Male to female ratios of the suspected (*n* = 562) and confirmed (*n* = 73) cases were 5.3:1 and 5.1:1 respectively. Of the 98 tested samples from the suspected cases, 25 tested negative for anti-HAV IgM antibodies. These included a child, three adolescents, 15 young adults and six adults (⩾40 years). There were three deaths among the laboratory-confirmed hepatitis A cases (4.1%) (3/73) in the age group of 30–45 years and these individuals did not have any co-morbid conditions.

The confirmed hepatitis A cases (*n* = 73) showed average nearest neighbour ratio of 0.11 with a *Z*-score of −14.53, indicating a significant clustering of the cases (*n* = 60) in ward nos. 13 and 11 (Fig. 1). The common symptoms in the confirmed hepatitis A cases included, yellow eyes (100%), fever (93.2%), vomiting (84.9%), nausea (54.8%), loss of appetite (43.8%), abdominal pain (34.2%), dark urine (26%), myalgia (23.3%), arthralgia (5.5%), fatigue (5.5%), clay coloured stool (2.7%) and diarrhoea (2.7%). Analysis of the alanine aminotransferase levels in the 23 confirmed hepatitis A cases showed a mean of 2340.1 ± 1091.5 IU/l and the levels ranged from >3000 IU/l (*n* = 5), 2001–3000 IU/l (*n* = 9), 1000–2000 IU/l (*n* = 6) to <1000 IU/l (*n* = 2). Serum bilirubin levels were also found to be elevated in 21/23 (91.3%) of these cases with a mean of 5.8 ± 2.8 mg/dl, while alkaline phosphatase levels were elevated in all 23 cases with a mean of 341.1 ± 144.9 IU/l.

**Fig. 2.**
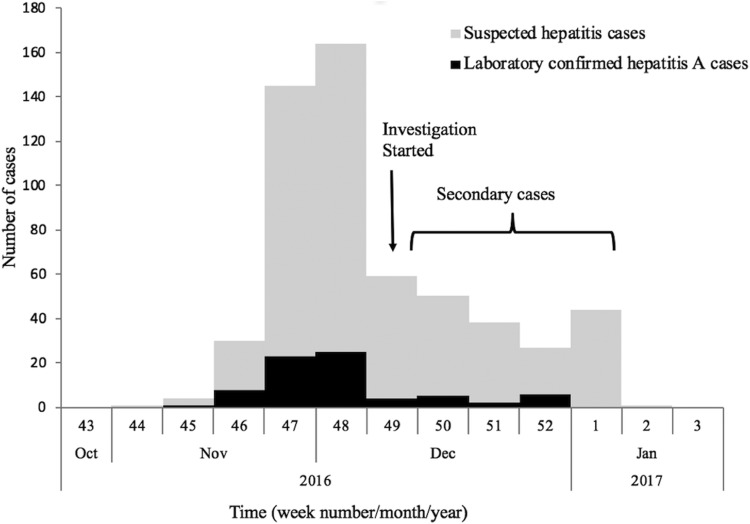
Weekly distribution of the suspected and laboratory-confirmed hepatitis A cases in the study area in Ernakulam district, Kerala, from October 2016 to January 2017.

The first case (35-year male) was reported on the 12th November from Nellikuzhi. He gave a history of visiting a new restaurant on its inaugural day, 24th October 2016, in Nellikuzhi. Of the suspected 562 cases, 86.1% (484/562) gave history of consumption of food or water or cold drinks from the same restaurant, located in ward no. 13 of Nellikuzhi (Fig. 1). The restaurant used water from a private well situated in its premise without any pre-treatment. A single peak of the cases was observed in the weeks 47 and 48 (Fig. 2).

### Environmental findings

It was noted that the suspected well was closely situated near a drainage line and did not have proper sanitary conditions in the surroundings. It was suspected that the well water probably got contaminated due to seepage of contaminated water. However, we were not able to confirm the presence of virus in the water since health authorities had super-chlorinated and sealed the well after suspecting association of the restaurant with hepatitis cases.

### Detection of HAV RNA from samples and virus genotype analysis

**Fig. 3.**
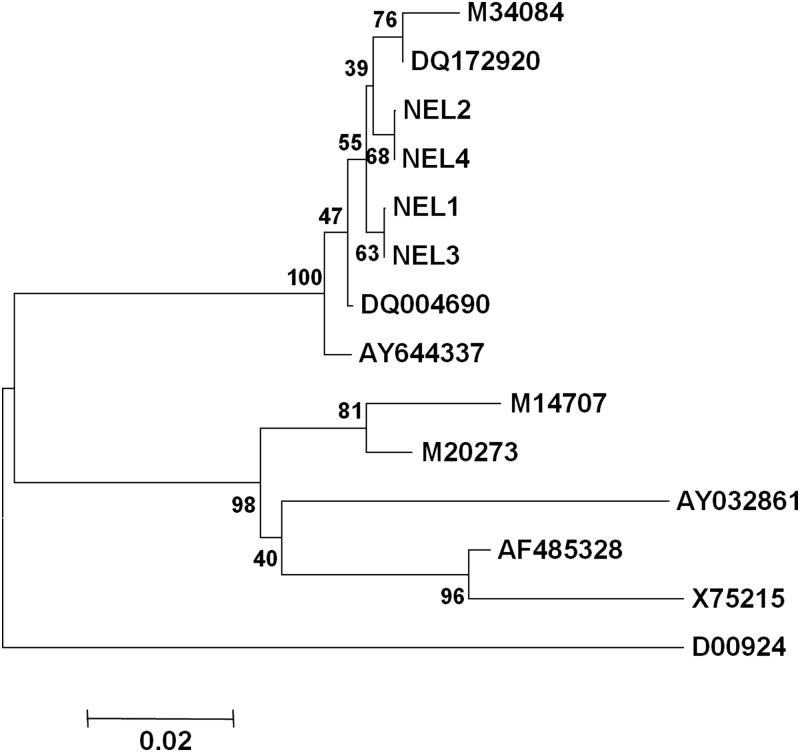
Phylogenetic analyses based on 5′NCR (291 nt) sequences of HAV isolates: the Nellikuzhi sequences are labelled as NEL1, NEL2, NEL3 and NEL4. Per cent bootstrap support is indicated by the values at each node. Accession numbers and strains of the sequences used for the analysis were as follows: genotype IA: X75215 (GBM), AF485328 (LY6), genotype IB: M14707 (HM175), M20273 (MBB), genotype IIIA: AY644337 (isolate from Germany), M34084 (PA21), DQ004690 (isolate from Kottayam, Kerala, India), DQ172920 (isolate from Daund, Maharashtra, India), genotype V: D00924 (AGM27) and Genotype VII: AY032861 (SLF88).

Of the nine stool samples collected from anti-HAV IgM positive cases, seven samples tested positive for HAV RNA. None of the samples tested positive for HEV RNA. Sequencing and phylogenetic analyses revealed the presence of genotype IIIA HAV in all the samples (Fig. 3). All water samples tested negative for HAV and HEV RNA.

### Prevention and control measures

The local health authorities did super-chlorination of all public wells in the Nellikuzhi area in December 2016 and created awareness among the people via posters, banners and mass media. The restaurants not having the Food and Drug Administration (FDA) permission and registration in the local panchayat offices were closed down.

### Serosurvey in the affected and unaffected area

A total of 110 blood samples were collected from apparently healthy individuals in the affected area, Nellikuzhi, which included children (*n* = 27), adolescents (*n* = 14), young adults (*n* = 33) and adults (*n* = 36) aged ⩾40 years. Of the 27 children aged ⩽9 years, 11% (3/27) were detected positive for anti-HAV IgG antibodies. Comparative data for the children from Ayavana could not be obtained as blood samples could not be collected from healthy children. Hence, for the comparison between affected and non-affected areas, only those age groups were considered for which samples could be collected from both the areas. In Nellikuzhi, 62.5% (52/83) individuals tested anti-HAV IgG positive and 19.3% (16/83) anti-HEV IgG positive. Eleven individuals were both anti-HAV and anti-HEV IgG positive. Overall, 68.7% (57/83) individuals had past exposure to either HAV and/or HEV in Nellikuzhi (Table 1).

**Table 1.** Serosurveys in hepatitis A outbreak area and non-affected area for detecting past infections of hepatitis A and hepatitis E

Category (area name)	Age group (years)	Total persons (*n*)	Serosurvey persons past exposure for HAV and HEV (%)			
			Anti HAV IgG and anti HEV IgG positive	Anti HAV IgG positive	Anti HEV IgG positive	Anti HAV IgG and anti-HEV IgG negative
Hepatitis affected (Nellikuzhi)	10–19	14	1 (7.1)	2 (14.3)	0	11 (78.6)
	20–39	33	1 (3.0)	15 (45.5)	4 (12.1)	13 (39.4)
	40–59	24	5 (20.8)	16 (66.7)	1 (4.2	2 (8.3)
	⩾60	12	4 (33.3)	8 (66.7)	0	0
	Total	83	11 (11.3)	41 (49.4)	5 (6.0)	26 (31.3)
Hepatitis unaffected (Ayavana)	10–19	8	0	0	1 (12.5)	7 (87.5)
	20–39	29	1 (3.4)	11 (37.9)	4 (13.8)	13 (44.8)
	40–59	37	8 (21.6)	23 (62.2)	1 (2.7)	5 (13.5)
	⩾60	26	10 (38.5)	16 (61.5)	0	0
	Total	100	19 (19.0)	50 (50.0)	6 (6.0)	25 (25.0)

In Ayavana, of the 100 healthy individuals, 69% (69/100) tested positive for anti-HAV IgG and 25% (25/100) tested positive for anti-HEV IgG. Nineteen individuals had both anti-HAV and anti-HEV IgG antibodies. Overall, 75% (75/100) individuals had past exposure to either HAV and/or HEV in Ayavana. On doing statistical analysis it was noted that there was no significant difference between past exposures to HAV/HEV in these two populations (Table 1).

## Discussion

The current study confirmed hepatitis A outbreak in Nellikuzhi, Ernakulam District, Kerala, India, in which a large number of cases among adults were traced back to one event. This suggests that India is going through epidemiological transition with respect to HAV infection. The suspected source of the virus was food or cold drinks served during the inaugural function of the restaurant in Nellikuzhi, as majority (86.1%) of the cases gave history of consuming either food or cold drink from the restaurant. Rapid rise of hepatitis cases in the weeks 47 and 48, followed by a fall 49 week onwards indicated possible point source outbreak. The cases at the tail end of the outbreak were possibly secondary cases (Fig. 1), reflecting community wide outbreak. It was also likely that the source of contamination at the restaurant continued till it was found to be associated with the outbreak. Laboratory confirmed that hepatitis A cases showed significant clustering in the region of ward no. 13, Nellikuzhi, where the restaurant was located (Fig. 1).

Majority (67.6%) of the suspected cases belonged to the age group of young adults indicating that substantial proportion of this age group was not exposed to HAV prior to this outbreak. With considerably lower seropositivity (~11%) seen amongst the children in Nellikuzhi, it can be speculated that HAV was not in circulation in the recent past in this area and was accidentally introduced there. Serosurveys done in the affected and unaffected areas may not represent the population of interest and therefore may introduce a source of bias as a convenience sample method (a type of non-probability sampling) where the sample is taken from a group of people easy to contact or to reach. However, in the outbreak situation due to limited resources, no other sampling method was feasible except for the persons who were available and willing to participate in the serosurveys.

The possible hypothesis of common source of the outbreak could have been tested by conducting analytical studies such as the case control study. However, it could not be done due to language barrier, requirement of ethical permissions in short time and limited staff. Also, we focused mainly on characterising the outbreak by time, place and person distribution. However, Rakesh *et al*. [8] have reported findings of their case control study and association of consumption of drinks/food from the hotel with the cases, confirming that this outbreak was a common source outbreak.

In the current outbreak, majority of cases were adults/young adults (~81%) and not children because they constitute major proportion of working population and more likely to consume outside food. Other possibility was that, children were also infected and probably missed as they remained asymptomatic. Older persons were spared since they had protective antibodies.

Kerala is one of the economically developed states in India, with high literacy rates (95%) and advanced in terms of social development and often compared with other developed countries. With better hygiene and standard of living in Kerala, children seem to escape infection in early childhood. A significant decline (<10%) in anti-HAV seropositivity in children below 5 years has been reported from Kerala [9]. Several hepatitis A outbreaks as well as increase in the number of sporadic hepatitis A cases in young adults and as many as 22 deaths have been reported from Kerala [2–4, 6, 10–12]. In the present hepatitis A outbreak, three deaths were reported.

It is important to note that a substantial out of pocket expenditure on the treatment of hepatitis A disease is a common problem in Kerala. It is noted that community-wide outbreaks are often prolonged and difficult to control and persist for 6–18 months [5]. Almost 70% of the population in Kerala is dependent on private health services. It is evident from the public health research findings and social media reports that there is a consistent demand of introducing hepatitis A vaccination in the population in Kerala considering the transition of hepatitis A epidemiology in India specifically among the population in Kerala. However, National Technical Advisory Group on Immunization (NTAGI), who advocates vaccine policy to Ministry of Health and family Welfare, Government of India, has recommended the use of hepatitis A vaccination only in the context of epidemic control and for individual use [13]. However, NTAGI has also indicated need of doing cost effectiveness studies on hepatitis A vaccination in the Indian population [13].

Hepatitis A outbreaks occur mostly during the rainy season, from June to November, probably due to heavy rains and floods in Kerala [3]. The present outbreak also occurred in the rainy season. Majority (80%) of households in the rural part of Kerala use well water for their domestic needs. In the absence of adequate sewerage network in rural areas, people use septic tanks for sewage disposal which leads to soil contamination. Since HAV is a stable virus it can persist for a long period in soil [14] and water logging during monsoon season probably helps the virus to percolate along with water to contaminate wells. Hence, there is a need to educate people about simple preventive measures while using groundwater during the rainy season. It is noteworthy that the reporting of increase in hepatitis A cases by the tertiary care hospital to local health authorities helped to initiate necessary control and preventive measures and awareness in the public during this outbreak. The prompt action against the new restaurant and closing of other unregistered eateries in the adjoining areas by the local government authorities were praiseworthy.

This outbreak was due to genotype IIIA HAV, as analysed from the seven HAV RNA positive stool samples of the confirmed hepatitis A cases (Fig. 3). Genotype IIIA is the predominant HAV genotype circulating in most parts of India [15–18]. Though there was a significant association between the confirmed cases and the restaurant, we could not confirm the virus source due to prior super-chlorination of the well water and unavailability of the food and cold drink samples. We attempted to detect virus by screening water samples from neighbouring households in the affected area, ice samples from the local market and soil samples from the area surrounding the suspected well, none were found positive for HAV RNA. This signifies the need of timely investigation of the outbreak along with laboratory support and preservation of suspected water/food samples in cold chain for identifying the source of infection.

## Conclusions

In conclusion, a large number of hepatitis A cases was found to be linked to a point source in the current outbreak. HAV infections are often symptomatic in young adults and likely to be seen in outbreak forms. There are several reports from India documenting an increase in adult HAV infections [19–22]. Kerala being an important tourist destination, disposal of waste and management of water sources are very critical. Considering increasing HAV naive population in Kerala, accidental introduction of the virus may have a huge toll on the health economy. Hepatitis A is a vaccine-preventable disease and several countries have documented lowering of hepatitis A incidence in the vaccinated cohorts and also in the general population due to herd immunity [23]. An occurrence of multiple HAV outbreaks in Kerala suggests that there is a need of evaluating hepatitis A vaccination in high-risk community by undertaking cost-effective studies. Such studies would generate evidence which would help policymakers for taking decision on hepatitis A vaccination in the region. Hepatitis A vaccine in high-risk groups in Kerala may be considered on priority so as to avoid such large scale outbreaks.

## References

[1] Koff RS (1992) Clinical manifestations and diagnosis of hepatitis A virus infection. Vaccine 10(Suppl 1), S15–S17. 1335649 10.1016/0264-410x(92)90533-p

[2] Arankalle VA (2006) Molecular characterization of hepatitis A virus from a large outbreak from Kerala, India. The Indian Journal of Medical Research 123, 760–769. 16885597

[3] Kumar T (2015) Viral hepatitis surveillance-India, 2011–2013. Morbidity and Mortality Weekly Report 64, 758–762. 26203629 10.15585/mmwr.mm6428a3 PMC4584861

[4] Rakesh PS and Sreelakshmi MK (2017) 84 outbreaks of Hepatitis A in last five years in Kerala state-are we resigning to fate? National Journal of Research in Community Medicine 6, 267–270.

[5] Rakesh PS (2014) Investigating a community-wide outbreak of hepatitis A in India. Journal of Global Infectious Diseases 6, 59–64. 24926165 10.4103/0974-777X.132040 PMC4049041

[6] Raveendran S (2016) Investigation of an outbreak of hepatitis A in a coastal area, Kerala, Southern India. Journal of Primary Care & Community Health 7, 288–290. 10.1177/2150131916647007 PMC5932701 27257046

[7] Arankalle V (2007) Evaluation of human (genotype 1) and swine (genotype 4)-ORF2-based ELISAs for anti-HEV IgM and IgG detection in an endemic country and search for type 4 human HEV infections. Journal of Viral Hepatitis 14, 435–445. 17501765 10.1111/j.1365-2893.2006.00801.x

[8] Rakesh PS (2018) Investigating a community wide outbreak of hepatitis A in Kerala, India. Journal of Family Medicine and Primary Care 7, 1537–1541. 10.4103/jfmpc.jfmpc_127_18 PMC6293930 30613555

[9] Rakesh PS (2017) Do we need to consider universalising the hepatitis A vaccine in Kerala, India? Journal of Health Systems 2, 1–2.

[10] Rakesh PS (2017) Out-of-pocket expenditure due to hepatitis A disease: a study from Kollam district, Kerala, India. The Indian Journal of Medical Research 146, 426–429. 29355152 10.4103/ijmr.IJMR_275_16 PMC5793480

[11] Arankalle VA (2004) Hepatitis A vaccine strategies and relevance in the present scenario. The Indian Journal of Medical Research 119, iii–vi. 15218976

[12] Mathur P and Arora N (2008) Epidemiological transition of hepatitis A in India: issues for vaccination in developing countries. The Indian Journal of Medical Research 128, 600–704. 19246792

[13] Minutes of the meeting of the National Technical Advisory Group on Immunization, India, Ministry of health and Family Welfare, Government of India, Nirman Bhavan, New Delhi, December 9, 2016.

[14] Parashar D, Khalkar P and Arankalle V (2011) Survival of hepatitis A and E viruses in soil samples. Clinical Microbiology and Infection 17, E1–E4. 10.1111/j.1469-0691.2011.03652.x 21939469

[15] Barde P (2014) Circulation of hepatitis A genotype IIIA virus in paediatric patients in central India. The Indian Journal of Medical Research 139, 940–944. 25109730 PMC4165008

[16] Chobe L and Arankalle V (2009) Investigation of a hepatitis A outbreak from Shimla, Himachal Pradesh. The Indian Journal of Medical Research 130, 179–184. 19797816

[17] Gurav YK (2015) Outbreak of hepatitis A in an orphanage in Pune city, India. International Journal of Tropical Medicine and Public Health 5, 1–4.

[18] Chadha M (2009) Outbreaks of hepatitis A among children in western India. Transactions of the Royal Society of Tropical Medicine & Hygiene 103, 911–916. 19155033 10.1016/j.trstmh.2008.11.014

[19] Acharya S (2006) Viral hepatitis in India. The National Medical Journal of India 19, 203–217. 17100109

[20] Hussain Z (2006) Increasing trend of acute hepatitis A in north India: need for identification of high-risk population for vaccination. Journal of Gastroenterology and Hepatology 21, 689–693. 16677154 10.1111/j.1440-1746.2006.04232.x

[21] Sharma A (2016) Upsurge in vaccine preventable hepatitis A virus infection in adult patients from a tertiary care hospital of North India. International Journal of Infectious Diseases 45, 457.

[22] Das K (2000) The changing epidemiological pattern of hepatitis A in an urban population of India: emergence of a trend similar to the European countries. European Journal of Epidemiology 16, 507–510. 11049092 10.1023/a:1007628021661

[23] Andre F (2006) Universal mass vaccination against hepatitis A. In: Mass Vaccination: Global Aspects-Progress and Obstacles Current Topics in Microbiology and Immunology 304, 95–114. 16989266 10.1007/3-540-36583-4_6

